# Novel Antioxidant Tripeptide “ACQ” Can Prevent UV-Induced Cell Death and Preserve the Number of Epidermal Stem Cells

**DOI:** 10.1155/2015/359740

**Published:** 2015-06-25

**Authors:** Hye-Ryung Choi, Jung-Won Shin, Jung-Im Na, Kyung-Mi Nam, Hyun-Sun Lee, Kyoung-Chan Park

**Affiliations:** Department of Dermatology, Seoul National University Bundang Hospital, 166 Gumi-ro, Bundang-gu, Seongnam-si, Gyeonggi-do 463-707, Republic of Korea

## Abstract

We found that tripeptide “ACQ: alanine-cysteine-glutamine” has significant DPPH scavenging activity compared to that of glutathione. Antioxidant effects of ACQ were tested in *in vitro* and *in vivo* models. When treated with H_2_O_2_, mock treated fibroblasts and keratinocytes showed strong staining by H_2_DCFA. But, ACQ showed good protective effects against hydrogen peroxide treatment. When mice were fed for 2 or 4 weeks, similar protective effects were observed. In the control group, epidermis was severely damaged by UV irradiation and apoptotic keratinocytes were observed. There were also numerous TUNEL positive cells. But in the ACQ group, epidermis became thicker and there was no sign of severe damage. Interestingly, the number of p63 cells was also higher in ACQ fed mice. To confirm the stem cell rescuing effects of ACQ, three-dimensional skin samples were constructed. Results showed that ACQ increased the expression of integrin *α*6 and the number of p63 positive cells. These findings showed that ACQ has good antioxidant activity and may increase stem cell activities by the regulation of integrin *α*6.

## 1. Introduction

The skin is composed of epidermis, dermis, and subcutaneous fat layer. Epidermis, multilayered tissue of mainly keratinocytes, is a continually renewable tissue supported by proliferation of keratinocyte stem cells. For the survival of stem cells, stem cell niche was proposed as a stem cell specific microenvironment and it would provide a condition that determines the stem cell number and stem cell mobility [1]. Extracellular matrix (ECM) may be a key component of the stem cell niche [2] and epidermal stem cells can directly contact the ECM because these cells express high levels of integrins. In addition, growth factors or mechanobiological factors also can affect stem cell fate [2, 3]. Furthermore, it is known that low oxygen tensions (hypoxia) maintain undifferentiated nature of stem cell phenotypes and also influence the proliferation and the fate of stem cells [4]. Thus, reactive oxygen species (ROS) regulate stem cell differentiation and we reported that redox status can affect the stemness and the proliferative potential of epidermal basal cells by modulating the microenvironment [5]. In addition, ROS enhance differentiation of human embryonic stem cells into mesendodermal lineage [6] and coenzyme Q10 can protect against hypoxia in neural stem cells [7].

These findings showed that free radical is very important in the regulation of stem cells. Skin is located at the outer layer of the human body and it can be a good model to study the effects of many biological agents. In this study, we tried to discover an effective antioxidant to protect skin from UV irradiation and to observe whether this antioxidant can rescue skin stem cells. By peptide library screening, we found that tripeptide “ACQ: alanine-cysteine-glutamine” has good antioxidant activity compared to that of glutathione (GSH). In this study, we present antioxidant effects of ACQ in* in vitro* and* in vivo* models and its beneficial effects on skin stem cells in three-dimensional skin equivalent models.

## 2. Materials and Methods

### 2.1. Reagents

Antibodies that recognize p63 (sc-8343) and integrin *α*6 (sc-6597) were purchased from Santa Cruz Biotechnology (Santa Cruz, CA, USA). Alexa Fluo488 chicken anti-rabbit IgG (H + L) (A21441, Molecular Probes) and Alexa Fluo555 donkey anti-goat IgG (H + L) (A21432, Molecular Probes) were obtained from Invitrogen (Carlsbad, CA, USA). Epidermal growth factor (EGF) was also obtained from Invitrogen (Carlsbad). Normal goat serum (5%, S-1000, VECTOR, Burlingame, CA, USA) was used for blocking. Diphenyl-2-picrylhydrazyl (DPPH), 2′,7′-dichlorofluorescin diacetate (H_2_DCFDA), bovine serum albumin (BSA), and H_2_O_2_ were obtained from Sigma-Aldrich Co. (St. Louis, MO, USA). Keratinocyte growth medium (KGM) and keratinocyte basal medium (KBM) were obtained from Lonza (Basel, Switzerland). Dulbecco's modified Eagle's medium (DMEM, LM001-05) was obtained from WelGENE (Daegu, South Korea). Fetal bovine serum (FBS, HyClone) was from ThermoScientific (Logan, UT, USA) and 4′,6-diamidino-2-phenylindole (DAPI, 10236276001) was obtained from Roche (Basel).

### 2.2. DPPH Assay of ACQ

Tripeptide “ACQ” was synthesized by Genescript (Hong Kong, China). To measure the antioxidant effects of ACQ and GSH, DPPH assay was used. DPPH is a stable free radical molecule which is commonly used in antioxidant assay. Two concentrations were used and each sample of stock solution (2 *μ*L of 100x) was added to 80 *μ*L of 0.25 mM DPPH and 118 *μ*L of 70% ethanol, to produce a final DPPH concentration of 0.1 mM. The mixture was vigorously shaken and left to stand in the dark, and its absorbance was measured at 517 nm using an ELISA reader (TECAN, Salzburg, Austria). GSH was used as the control.

### 2.3. Culture of Normal Human Cells and Effects of ACQ

Normal human fibroblasts and keratinocytes were isolated from human foreskins obtained during circumcision [8]. All samples were obtained with informed consent. All experiments were carried out using cells between 3rd to 6th passages. Fibroblasts were cultured in DMEM (LM001-05, WelGENE) supplemented with 10% FBS (ThermoScientific), 1 mM sodium pyruvate, 50 *μ*g/mL streptomycin, and 50 *μ*g/mL penicillin at 37°C in 5% CO_2_. Keratinocytes were cultured in KGM (Lonza). The media were changed every 2 or 3 days.

To test the cytotoxic effects on fibroblasts, 2,000 or 4000 cells were seeded into each well of 96 well. After 24 hrs, medium was replaced with serum free DMEM (WelGENE) and incubated for another 24 hrs. Then, cells were tested with increasing doses of ACQ for 24 hr (toxicity) or 72 hrs (proliferation assay). To test the effects on keratinocytes, 10^4^ cells were seeded into each well of 96-well plates by using KGM (Lonza). After 24 hrs, medium was replaced with starvation medium (KBM, Lonza) with 0.1% BSA (Sigma-Aldrich) and incubated for another 24 hrs. Then, increasing doses of ACQ were added and incubated for 24 hrs (cytotoxicity) or 72 hrs (proliferation). CCK-8 solution (100 *μ*L of 5 mg/mL, Cell Counting Kit-8, Dojindo, CD04-13, Kumamoto, Japan) was added and the plates were incubated for 2 hrs. Then, the optical density was determined at 400 nm using a SpectraMax plus 384 (Molecular Devices, #plus 384, Sunnyvale, CA, USA).

### 2.4. Effects of ACQ in Fibroblasts and Keratinocytes against H_2_O_2_

Normal human fibroblasts (4,000 cells) were seeded onto flat bottom 96-well TC-treated microplate (#3603, Corning, Lowell, MA, USA). After 24 hrs, medium was replaced with serum free DMEM (WeLGENE) containing ACQ (0~1 *μ*g). After an additional 24 hrs, medium was removed and 1x DPBS (Dulbecco's phosphate buffered saline) with or without H_2_O_2_ (H1009, Sigma-Aldrich) was added and incubated overnight. After removing DPBS solution, cells were once washed with DPBS. To measure the cellular antioxidant effects, DCF assay was used. In the presence of ROS, H_2_DCFDA is oxidized to DCF. So, DPBS with H_2_DCFDA (10 *μ*M, D6883, Sigma-Aldrich) was added and incubated for 30 min at 37°C in 5% CO_2_ incubator. After washing, cells were observed with fluorescent inverted microscope (Carl Zeiss, Axio Observer, Jena, Germany). Photographs were taken from 3 different dishes and fluorescence intensity was analyzed by Meta Imaging Series Software (MetaMorph Version 7,700, Molecular Devices, Downingtown, PA). For keratinocytes, 10,000 cells were seeded onto 96 wells (#3603, Corning). Then, the same procedure was done with fibroblasts.

### 2.5. Oral Supplementation of ACQ Protects against UVB Irradiation

Experimental protocols were approved by the Seoul National University Bundang Hospital (SNUBH) animal IRB. BALB/c nude mice were purchased from Daehan Biolink Co. (Chungbuk, South Korea). The animals were maintained according to animal care guidelines (20~22°C room temperature, 50 ± 10% relative humidity, and 12 h light/12 h dark cycle). Regular diet was supplied and ACQ was provided in sterile distilled water (1 mg/mL). The animals were randomly chosen and fed for 2 or 4 weeks ad libitum. Animals were irradiated with UVB (SANKYO DENKI, #G4T5E, Tokyo, Japan). The dose of UVB was quantified with a Waldmann UV meter (model number 585100: Waldmann Co., Villingen-Schwenningen, Germany) and 600 mJ/cm^2^ UVB was delivered. After 24 hrs, mice were killed by CO_2_ treatment and the dorsal skin was obtained. Skin samples were fixed in 10% phosphate-buffered formalin at 4°C for 24 h. The samples were then dehydrated in ascending concentrations of ethanol, cleared in xylene, and embedded in Paraplast (Oxford Labware, St. Louis, MO, USA). Serial sections were made and stained with Hematoxylin and Eosin (H&E). For TUNEL staining, DeadEnd Fluorometric TUNEL System (Promega, #G3250, Madison, WI, USA) was used following the manufacturer's recommendation. After the staining procedure, slides were stained with DAPI (1 *μ*g/mL). After mounting (VECTASHIELD Mounting Medium, H-1400, Vector Laboratories, Burlingame, CA, USA) and observed with confocal laser scanning microscope (Carl Zeiss, #LSM710 Jena, Germany). Immunohistochemical staining was also performed.

### 2.6. Reconstruction of SEs and the Effects of ACQ

Dermal substitutes were prepared by our previous method [8]. Human keratinocytes (1 × 10^6^ cells) were then seeded onto the dermal substitute. Next, the samples were cultured in a submerged state for 2 days, after which they were cultured at the air-liquid interface for 12 days. The growth medium consisted of DMEM and Ham's nutrient mixture F12 at a ratio of 3 : 1 supplemented with 5% FBS, 0.4 *μ*g/mL hydrocortisone, 1 *μ*M isoproterenol, 25 *μ*g/mL ascorbic acid, and 5 *μ*g/mL insulin. Low concentration of EGF (1 ng/mL, Invitrogen) was added during submerged state, while higher concentration of EGF (10 ng/mL) was added during the air-liquid interface culture condition. The medium was changed three times per week and all experiments were repeated at least twice under the same conditions. To test the effects of ACQ, two different concentrations of ACQ (0.1, 1 *μ*M) were added during the air-liquid interface culture conditions.

### 2.7. Histology and Immunohistochemistry

Finally, SEs were fixed in Carnoy's solution (ethanol/chloroform/acetic acid 6 : 3 : 1) for 30 min and then processed for conventional paraffin embedment. Briefly, 4 to 6 *μ*m thick sections were prepared and stained with H&E. Confocal microscopic examination was performed. To visualize p63, cells were treated with 0.2% Triton-X 100 (T8787, Sigma-Aldrich). After staining with DAPI (1 *μ*g/mL, 10236276001, Roche), image was analyzed by ZEN 2011 microscope software (Carl Zeiss).

### 2.8. Statistics

The data were tested with *t*-tests using Microsoft Excel (Microsoft Corporation, Seattle, WA).

## 3. Results

### 3.1. Antioxidant Activity of ACQ

**Figure 1:**
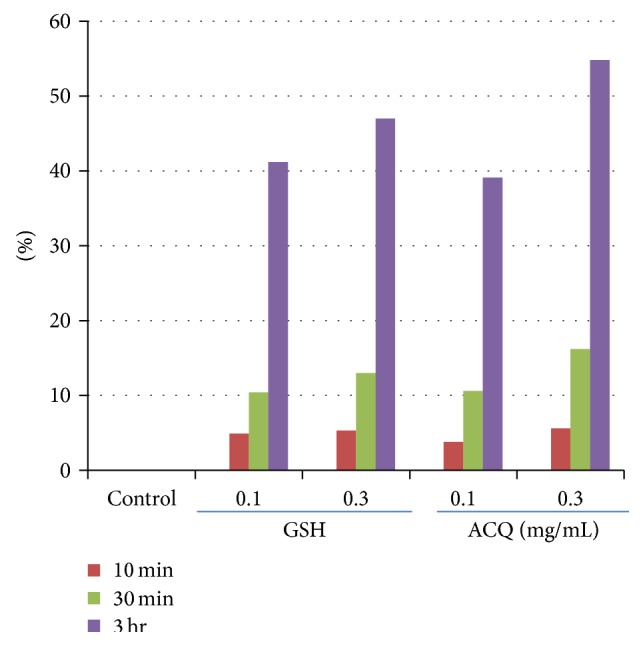
Scavenging effect of ACQ for DPPH. DPPH assay was done for 10 min, 30 min, and 3 hr, and GSH was used as control. The values shown are the means of triplicate wells. Experiment was performed 2 times.

Scavenging activity was tested by DPPH assay. At 10 min, antioxidant activity was less than 10%. However, these activities rose dramatically after 3 hrs in all conditions. In general, antioxidant activity of ACQ was similar to those of GSH (Figure 1).

### 3.2. Protective Effects of ACQ in Cultured Fibroblasts and Keratinocytes against Hydrogen Peroxide

**Figure 2:**
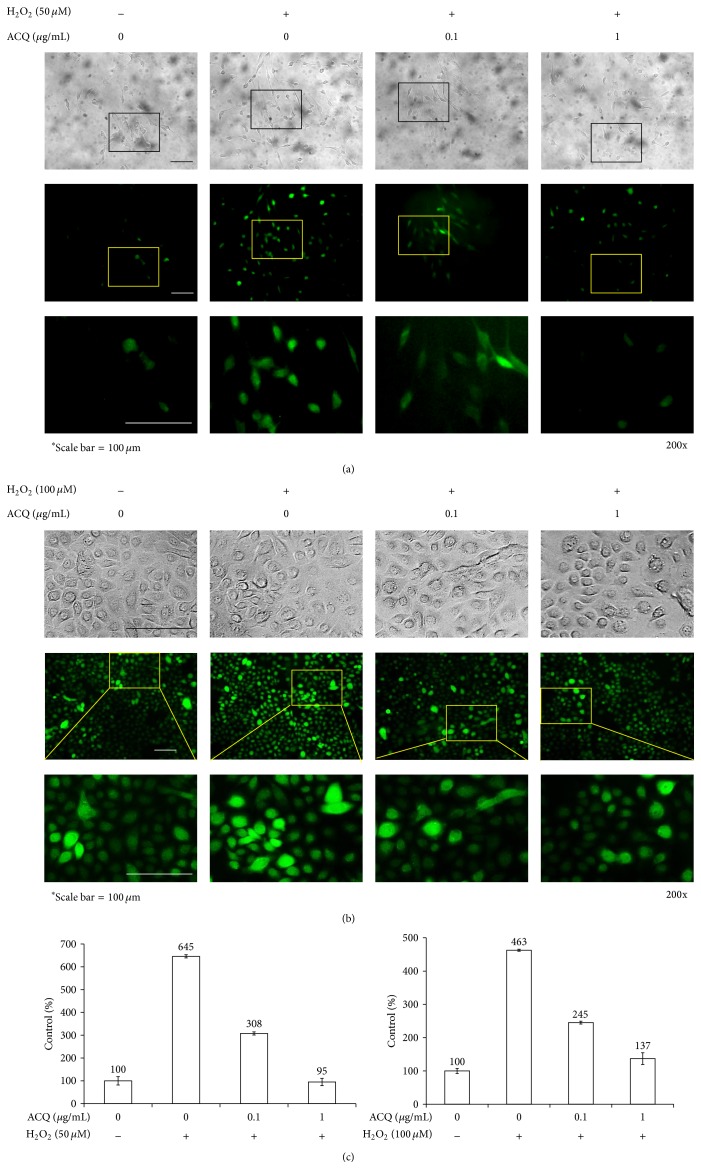
Protective effects of ACQ against hydrogen peroxide treatment. (a) Normal human fibroblasts or (b) normal human keratinocytes were incubated overnight with H_2_O_2_ after 24 hr treatment of ACQ. (c) Intensity of fluorescence (Lt: fibroblasts, Rt: keratinocytes). Experiment was repeated 2 times and representative data is shown (×200, scale bar is 100 *μ*M).

Cytotoxic or proliferative effects were tested between the concentration of 0~1 *μ*g/mL. In these concentrations, ACQ was not cytotoxic or proliferative to both fibroblasts and keratinocytes (data not shown). To generate free radicals in the cells, H_2_O_2_ was treated. Based on cytotoxicity data of ACQ, 50 *μ*M or 100 *μ*M was chosen for fibroblasts and keratinocytes, respectively (data not shown). Nontreated fibroblasts rarely showed positive staining by H_2_DCFA (D6883, Sigma-Aldrich). In contrast, H_2_O_2_ treated cells showed bright positive staining. However, addition of ACQ (0.1, 1 *μ*g/mL) effectively reduced the number of positively stained cells in fibroblasts (Figures 2(a) and 2(c)). Compared to fibroblasts, nontreated normal human keratinocytes showed relatively strong staining by H_2_DCFA. As expected, H_2_O_2_ (100 *μ*M) treatment dramatically increased the staining intensity by and this was decreased by ACQ treatment (Figures 2(b) and 2(c)).

### 3.3. UV Protective Effects in Nude Mice

**Figure 3:**
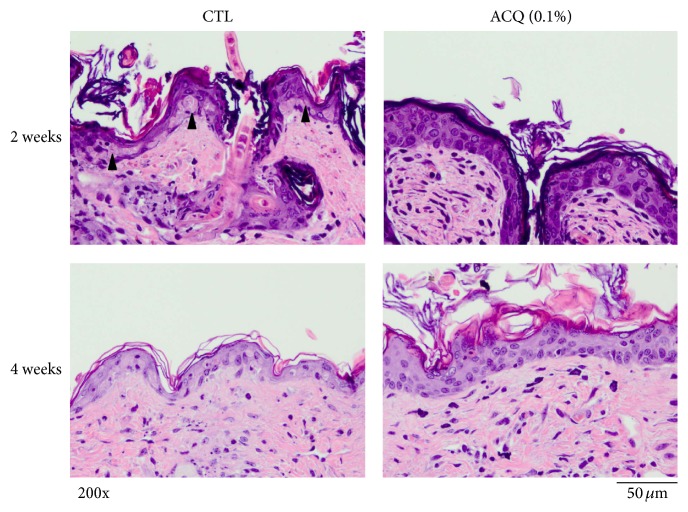
Protective effects by ACQ against UV irradiation. Control mice and ACQ fed mice (2 and 4 weeks) were irradiated with 600 mJ/cm^2^ of UVB. In control mice, epidermis became thinner and apoptotic cells were observed. But in ACQ fed mice, epidermis became thicker and severe damage was not observed. Experiment was repeated 2 times and representative data is shown (×200, scale bar is 50 *μ*M).

BALB/c nude mice were fed for 2 or 4 weeks. Each group (control mice, UV irradiated mice) consists of 2 mice and experiment was repeated 2 times. One day after UV irradiation, biopsy was taken. In the control group, thinning of epidermis was observed after UVB irradiation. In addition, several apoptotic keratinocytes were observed. But in the ACQ group, epidermis became thicker even after UVB irradiation. In addition, apoptotic cells were barely observed and these findings suggested that ACQ may have protective effects against UV irradiation (Figure 3).

**Figure 4:**
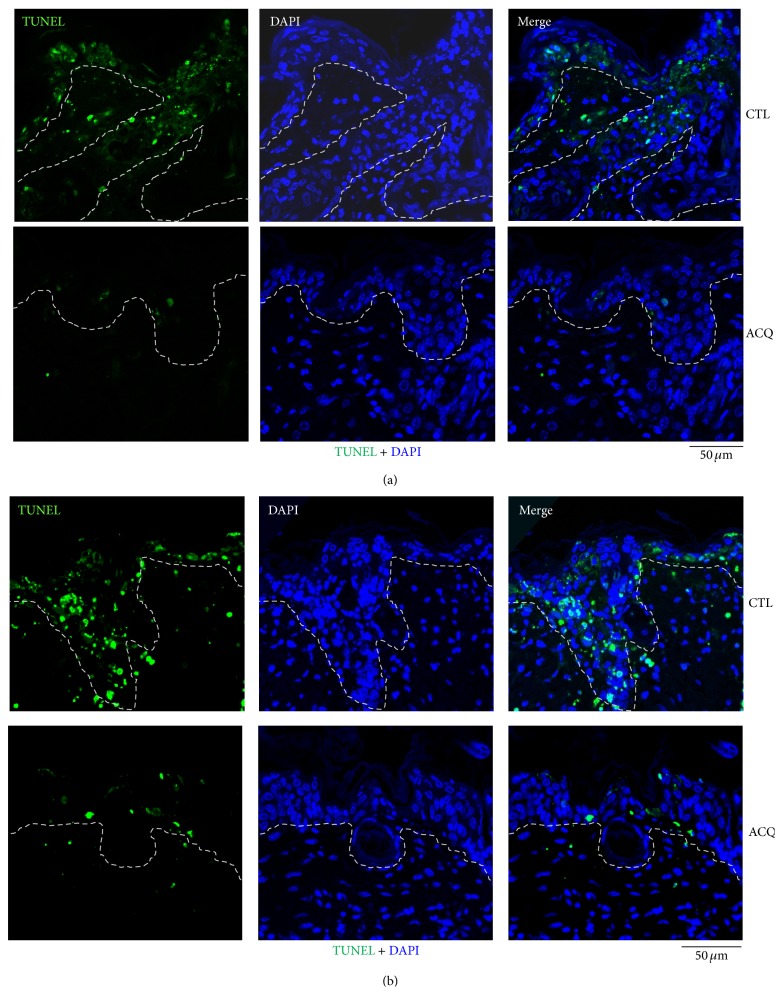
Decreased TUNEL positive cells in ACQ fed mice. After UVB irradiation (600 mJ/cm^2^), sections were stained for apoptotic cells. Compared to numerous TUNEL positive cells in control mice, ACQ fed mice rarely showed TUNEL positive cells (dashed line means basement membrane, ×200; scale bar is 50 *μ*M).

In order to visualize apoptosis, TUNEL staining was performed. Control group showed a lot of TUNEL positive cells, whereas only a few TUNEL positive cells were in the ACQ group (Figures 4(a) and 4(b)).

**Figure 5:**
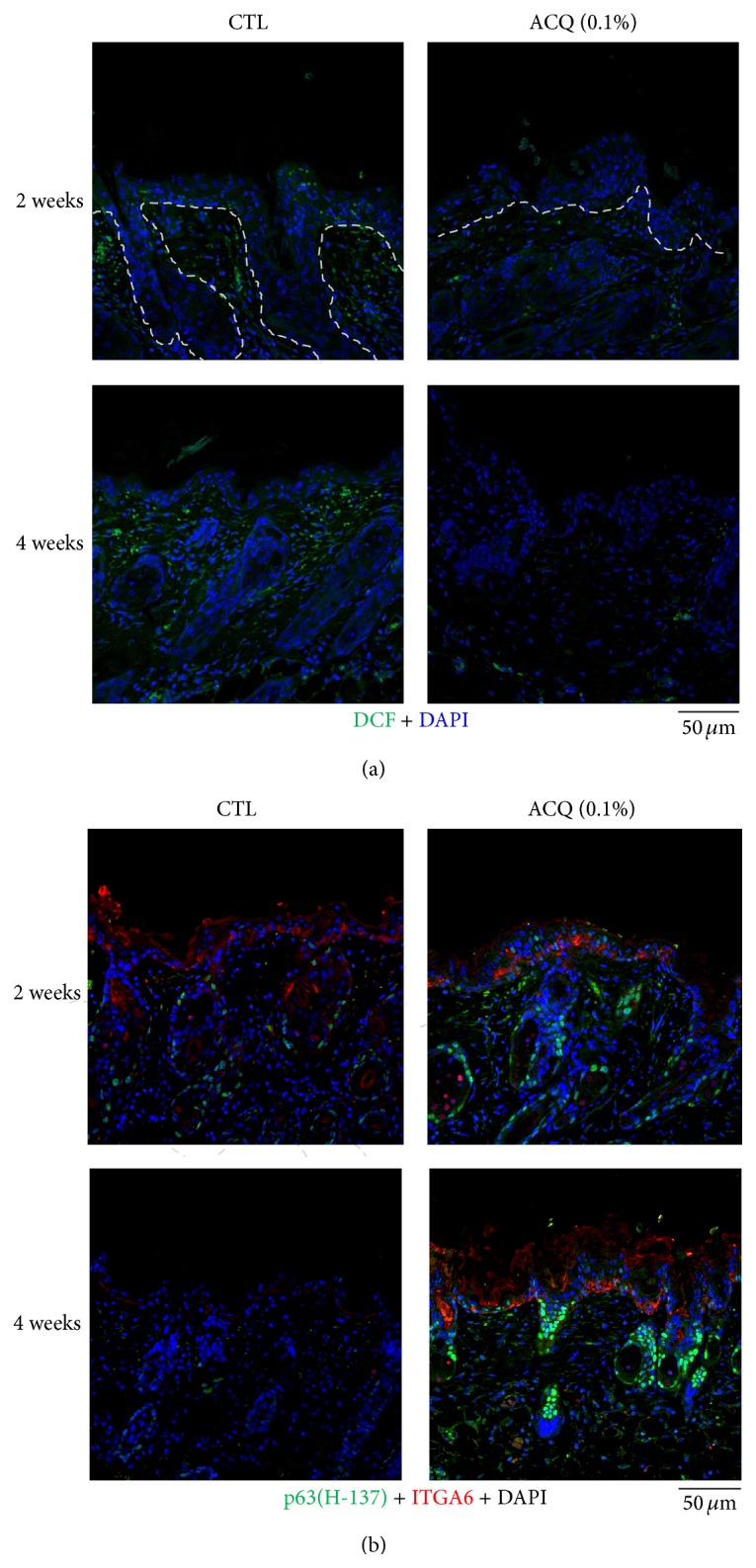
Decreased DCF staining and increased p63 positive cells in ACQ fed mice. (a) After UVB irradiation, control mice showed strong DCF staining but ACQ fed mice showed weak DCF staining (dashed line means basement membrane; scale bar is 50 *μ*M). (b) Confocal microscopic examination showed that there is strong signal of p63 and integrin *α*6 in ACQ fed mice compared to control mice. There was nonspecific staining of integrin *α*6 at the upper dermis (green; p63 staining, red; integrin *α*6 staining, ×200, scale bar is 50 *μ*M).

To visualize the free radicals, sections were stained with H_2_DCFA (Sigma-Aldrich, D6883) and results showed strong staining in control mice but these findings were not observed in ACQ treated mice (Figure 5(a)). Furthermore, the number of p63 positive cells was also higher in the ACQ group (Figure 5(b)).

### 3.4. Reconstruction of SEs and the Effect of ACQ

**Figure 6:**
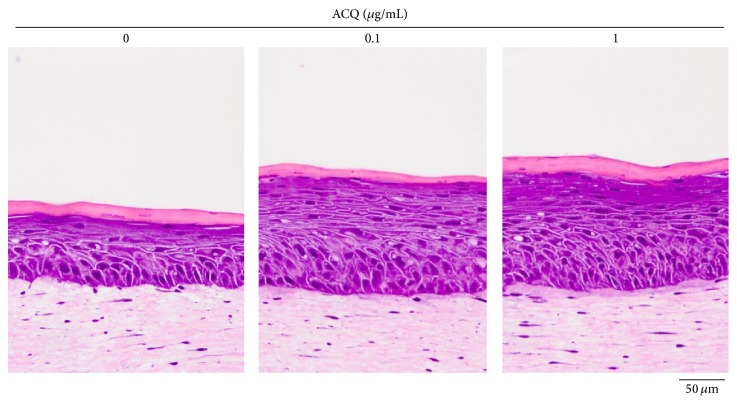
Histologic findings of skin equivalents. Compared to control SE, ACQ (0, 0.1, and 1 *μ*g/mL) treated SE became thicker. Experiments were repeated twice and the result shown is a representative experiment (×200, scale bar is 50 *μ*M).

**Figure 7:**
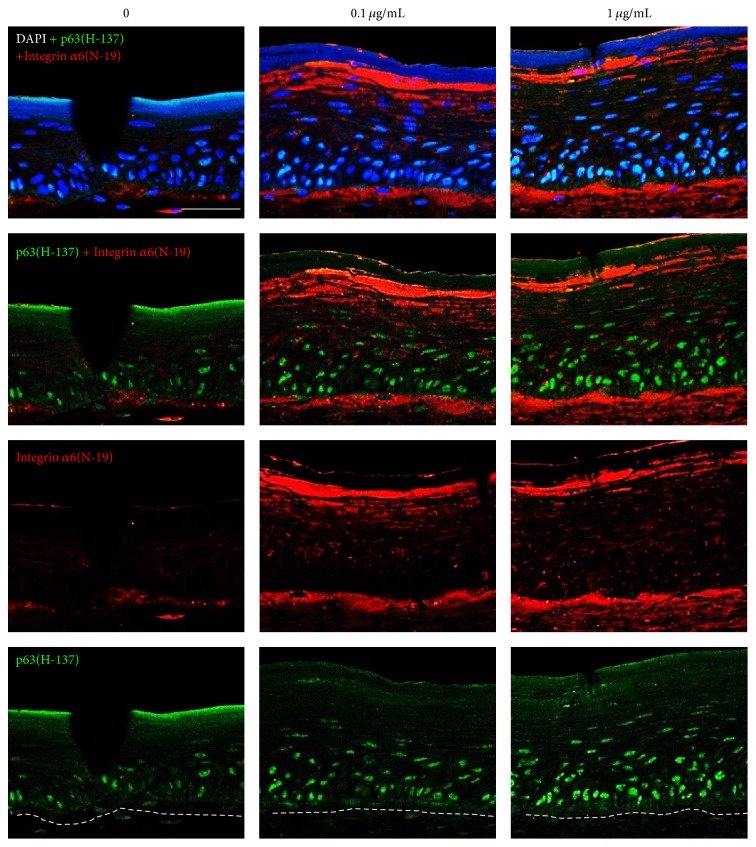
Confocal microscopic examination of skin equivalents. (a) The number of p63 positive cells increased significantly in response to treatment with ACQ compared to the control. In addition, the expression of *α*6 integrin was also significantly increased but nonspecific staining was observed at the upper epidermis (×200, scale bar is 50 *μ*M).

Results showed that epidermal thickness is dramatically increased by ACQ treatment by dose-dependent manner (0.1, 1 *μ*g/mL) (Figure 6). Increased thickness was correlated with the increased number of keratinocytes but differentiation was not affected. In addition, confocal microscopic examination showed increased number of p63 positive cells in ACQ treated samples. Furthermore, integrin *α*6 staining was definitely increased by ACQ treatment (Figure 7).

## 4. Discussion 

Oxidative stress damages cell structure and function by reactive oxygen-containing molecules and chronic excessive inflammation [9]. In order to control oxidation reactions, there are complex systems of multiple types of antioxidants, such as GSH, vitamin C, vitamin A, and vitamin E as well as enzymes such as catalase, superoxide dismutase, and various peroxidases [10]. GSH is a cysteine-containing peptide found in most forms of aerobic life [11]. It is synthesized in cells and has antioxidant properties since the thiol group is a reducing agent. In the body, GSH is one of the most important cellular antioxidants [11]. Specifically, thioredoxin and GSH are two of the major thiol-containing small compounds [12]. Thus, there is a possibility to find an alternative small molecular antioxidant compound which is beneficial in the body. In this study, we screened the cysteine-containing peptide library and found that ACQ has significantly good antioxidant activity. GSH is widely used as nutritional supplement in market. Thus, the effects of ACQ could be investigated in terms of nutritional supplement or medicinal effects. First of all, antioxidant effects were tested in cultured cell models. Fibroblasts and keratinocytes were cultured and treated with or without hydrogen peroxide and/or ACQ (Figure 2). Results showed that ACQ effectively protects fibroblasts and keratinocytes from the hydrogen peroxide stress. Interestingly, normal human keratinocytes showed stronger staining intensity by H_2_DCFA compared to fibroblasts (Figure 2(b)). It means that keratinocytes are under the high free radical stress even in normal condition. Second, antioxidant effects were tested in nude mice. Mice were fed for 2 or 4 weeks. Protective effects were observed with either 2 or 4 weeks feeding. By the intake of ACQ (0.1%) solution, epidermis became thicker after UV irradiation. H&E staining also showed that apoptotic cells are not abundant in ACQ treated mice (Figure 3). Furthermore, TUNEL positive cells were rarely observed in ACQ treated mice compared to control mice (Figure 3). All these findings suggested that ACQ intake is protective against UV irradiation and even 2-week supplementation is enough for the protective effects of ACQ in this study.

It has been reported that stem cells are much less susceptible to damage from environmental stresses such as hydrogen peroxide than differentiated cells [13]. Thus, free radical status was investigated by DCF staining (Figure 5(a)). Results showed that there is much less DCF staining in ACQ fed mice compared to control mice. In addition, the number of p63 positive cells is much higher in ACQ fed mice skin (Figure 5(b)). In order to verify stem cell enriching effects by ACQ, three-dimensional skin models were constructed. Results showed that ACQ treatment clearly increased the number of p63 positive cells along with the increased staining of *α*6 integrin (Figure 7). It is reported that endothelial progenitor cells are equipped with protection against oxidative stress compared to mature umbilical vein endothelial cells [13]. In addition, it is reported that antioxidant proteins and reactive oxygen species are decreased in a murine epidermal side population with stem cell-like characteristics [14]. Thus, free radical status should be closely related with stem cell environments. In this study, we found a novel tripeptide antioxidant which increased the epidermal thickness and number of p63 positive cells. In the skin, DNA labeling study and cell turnover studies demonstrated that long-lived stem cells were interspersed throughout the basal layer. However, the issue of stem cell marker in skin is still under debate [15]. Although the number of p63 positive cells is increased by ACQ treatment, ACQ may increase the proliferation of keratinocytes or delay late step of differentiation. By verifying more specific stem cell marker, exact effects of ACQ can be investigated in terms of stem cell fate. Peptides are chains of amino acids that can trigger various cellular processes [16]. Among these, short bioactive peptides are easier to deliver and several types of peptides have been reported to have diverse biological effects. However, the effects of short peptides are not well investigated. GSH is known to have antioxidant properties since cysteine moiety is a reducing agent [11]. It is a tripeptide with a gamma peptide linkage between the carboxyl group of the glutamate side-chain and the amine group of cysteine. Also, it is the major endogenous antioxidant produced by cells, participating directly in the neutralization of free radicals and reactive oxygen species, as well as maintaining exogenous antioxidants such as vitamins C and E in their reduced (active) forms. Thus, peptide antioxidant might be an antioxidant which acts like an endogenous antioxidant reservoir like GSH. By screening internal library, it was found that short tripeptide “ACQ” has good antioxidant effects. Interestingly, GSH and ACQ initially showed relatively weak antioxidant effects but showed delayed effects (Figure 1). Thus, these antioxidants can work as an endogenous reservoir of antioxidant systems.

Stem cells are localized in “niches”—specific sites that regulate their participation in tissue maintenance and repair [17]. The stem cells, whether embryonic or adult, are controlled by the interaction between intrinsic transcriptional regulations and extrinsic signals. In this study, three-dimensional skin models were constructed and treated with increasing concentrations of ACQ. Results showed that the number of p63 positive cell is increased by ACQ treatment (Figure 7). The concept of stem cell niche is important for the survival and fate of stem cells [1]. Stem cells receive extrinsic signals from the local microenvironment in which the stem cells are located. It is well known that ECM is an important niche component for stem cells. In the skin, higher levels of integrin were reported to be a marker of epidermal stem cells which could be an important index of stem cell fate [18, 19]. However, few studies have been performed to dissect how epidermal integrin and ECM gene expression is regulated. In our previous study, we showed that hair follicle dermal sheath cells increased the epidermal thickness and expression of *α*6 integrins [20]. By the following study, it was found that IGFBP-2 was a major factor from dermal sheath cells that regulates regenerative capacity of skin and plays an important role for stem cell characteristics in human epidermal keratinocytes [21]. IGFBPs have been reported to form biologically active complexes with IFG-1 and vitronectin (VN) and IGF-1 protects keratinocytes against UV-B damage [22, 23]. Moreover, these responses require activation of both the IGF receptor and the VN-binding *α*_*ν*_ integrins [22]. These findings suggested that IGF, IGFBP, and ECM proteins are key regulators in stem cell physiology and the levels of integrins may be an important factor in the maintenance of epidermal stem cells in the skin. Furthermore, activation of *β*1 integrin is reported to decrease terminal differentiation of cells [24]. Signaling studies demonstrated that ERK pathway downstream from *β*1 integrins is important in inhibiting differentiation signals [25]. In this study, *α*6 integrin is significantly increased by ACQ treatment (Figure 7). It means that ACQ acts as an antioxidant and could increase the levels of *α*6 integrin. These findings may construct a good microenvironment in which more stem cells can survive. Further study is necessary to find more interactions between antioxidants and stem cell behaviors.

## 5. Conclusions

A novel tripeptide “ACQ” was found to have significantly good antioxidant activity and these effects were clearly demonstrated by* in vitro* and* in vivo* animal models. Furthermore, stem cell enriching effects were observed in* in vivo* animal models and these effects were also shown in three-dimensional skin models. Immunohistochemical staining suggested that ACQ increases the number of p63 positive cells along with the increased expression of integrin *α*6.

## Abbreviations

ECM: Extracellular matrix
GSH: Glutathione
ROS: Reactive oxygen species.
